# Doppler Data Association Scheme for Multi-Target Tracking in an Active Sonar System

**DOI:** 10.3390/s19092003

**Published:** 2019-04-29

**Authors:** Yu Yao, Junhui Zhao, Lenan Wu

**Affiliations:** 1 School of Information Engineering, East China Jiaotong University, Nanchang 330031, China; junhuizhao@hotmail.com; 2 School of Information Science and Engineering, Southeast University, Nanjing 210096, China; wuln@seu.edu.cn

**Keywords:** Doppler data association (DDA), Doppler measurement, kinematic state estimation, multi-target tracking, tracking performance

## Abstract

In many wireless sensors, the target kinematic states include location and Doppler information that can be observed from a time series of range and velocity measurements. In this work, we present a tracking strategy for comprising target velocity components as part of the measurement supplement procedure and evaluate the advantages of the proposed scheme. Data association capability can be considered as the key performance for multi-target tracking in an active sonar system. Then, we proposed an enhanced Doppler data association (DDA) scheme which exploits target range and target velocity components for linear multi-target tracking. If the target velocity measurements are not incorporated into target kinematic state tracking, the linear filter bank for the combination of target velocity components can be implemented. Finally, a significant enhancement in the multi-target tracking capability provided by the proposed DDA scheme with the linear multi-target combined probabilistic data association method is demonstrated in a sonar underwater scenario.

## 1. Introduction

Besides position measurements, Doppler measurement can offer supplementary statistics about target state, which would enhance tracking performance [1]. The problem of multi-radar tracking using both position and radial velocity measurements was discussed in References [2,3]. The authors presented the track-while-scan algorithm of maneuvering targets in a clutter environment. The filtering algorithm was nonlinear and adaptive. The measurement of two or more different radial velocity components allows the calculation of rectangular velocity components [4]. The main problem for multi-target tracking is distinguishing between measured values resulted from a specific target and measured values caused by other radar target echoes or interference [5,6].

The Doppler measurements are employed in a couple with the target range information as supplementary state information, which is used to overcome this problem of recognizing range-overlapped targets. Literature [7] put forward an advanced joint probabilistic data association scheme, which utilizes Doppler measurements along with range measurements via a nonlinear programming method. Compared with the traditional joint probabilistic data association method, the tracking performance of the modified joint probabilistic data association algorithm is obviously improved for multi-target tracking in noisy jamming environment. Reference [8] developed a method by using an interacting multiple model estimator including several extended Kalman filter elements to deal with Doppler measurements. Several kinds of particle filters were used to deal with the component of target velocity measurements [9]. Similar methods have been presented in [2,8] as well (and references therein). The authors proposed that they have to employ nonlinear filters at the receiver to process the additional Doppler measurements.

From References [10,11], the error cross-correlation between transformed range measurements and Doppler measurements has been addressed. The two-step optimal estimator is another kind of sequential filtering technique, which was employed for multi-target tracking which comprises a Doppler measurements procedure [12]. Reference [13] presented a sequential filtering algorithm to improve the performance of multi-target tracking. However, the approach was heavily dependent on the use of extended Kalman filters for calculating the target estimated and predicted state. If target velocity measurements are involved as part of the target state vectors, the interacting multiple model estimators must be employed to deal with the nonlinearity distortion between range and velocity measurement. The error of observation between location and velocity is correlated. The error is a key problem when the velocity component is to be integrated in the sensor system to enhance multi-target tracking capability. Reference [14] developed other types of nonlinear filters, for example the particle filters or unscented Kalman filters, which are employed to replace the extended Kalman filters for an improved performance. References [15,16] discussed the problem of joint detection and tracking of a target using multi-static Doppler-only measurements. The authors developed for this application of a multi-sensor Bernoulli particle filter with information gain-driven receiver selection. A consensus dual-stage nonlinear filter algorithm was presented to solve the Doppler-only target tracking problem in a distributed and scalable way [17]. Based on such a decomposition, a novel dual-stage filter for centralized multi-sensor Doppler-only tracking was proposed [18]. Reference [19] developed a consensus Gaussian mixture cardinalized probability hypothesis density filter to distributed multi-target tracking with range and Doppler sensors.

However, the supplementary computational load is a challenging problem. One of many thorny issues for multi-target tracking is distinguishing between observations achieved by a target of interest and observations derived from the jamming targets or interference noise. Reference [20] presented the data association problem for the multi-target tracking. Then, the target velocity measurements were employed in alliance with the range component as a supplementary discriminant of observation derived from overcoming the issue of discriminating dense objects [21]. Actually, among all the currently existing methods stated above, a remarkable attainment is the enhanced performance in measurement supplement profited from the combination of velocity observations. 

Reference [22] developed hierarchical cognitive radar processing by applying the fully adaptive radar framework for cognition to a distributed radar network engaged in single target tracking. Two monostatic radar nodes are connected through a fusion center, and transmitted waveforms are adapted in real-time. However, the hardware requires the two radars to operate at the same pulse repetition frequency, limiting the degrees of freedom and affecting the velocity tracking accuracy. While previous research [23] has resulted in similar adaptive systems, this work presents a new approach to adaptive radar networks, treating each node as an independent instance of the framework for cognition. The authors in [24] formalized the work in [25,26] and presented a cognitive radar framework for a system engaged in target tracking. The model includes the higher-level tracking processor and specifies the feedback mechanism and optimization criterion used to obtain the next set of sensor data. The authors in [27] determined an optimal range for angle tracking radars based on evaluating the standard deviation of all kinds of errors in a tracking system. As the method increases in complexity, the usage of nonlinear filters might lead to system stability and capability problems. 

In this paper, in order to improve the performance of multiple extended target tracking, we analyze the advantage of combining target velocity measurements into the target state vectors, then present an advanced Doppler data association (DDA) scheme which employs target range and velocity components for improving target tracking performance. The principal difference between the proposed DDA scheme and existing methods is that in the former, the target velocity component is utilized only for data association. A joint likelihood for target velocity and range observations is used for the measurement supplement while potential influences on the multi-target tracking from the target velocity component might not be overlooked. However, the proposed scheme offers a robust usage of velocity observations that would realize significant performance enhancements to those stated in the literature without the supplementary computational burden due to the use of nonlinear filters [28].

A linear framework is utilized for multi-target tracking, which results in the application of an advanced linear multi-target (LM) integrated probabilistic data association (IPDA) technique [29,30,31,32,33]. By comparing the LM tracking algorithm and the advanced LM integrated Doppler measurements association algorithm, the superiority of the Doppler measurements association method is verified in a sea environment and the multi-target tracking capability of approaches using and without using the proposed method are compared. Both theoretical analysis and simulation results demonstrate that the proposed system based on the Doppler measurements association method has a great performance enhancement and less operation processing burden compared with the traditional method. 

The contributions of this paper can be summarized as follows:

(1) We present a tracking strategy for comprising target velocity components as part of the measurement supplement procedure and evaluate the advantages of the proposed method.
(2) We introduce a feasible scheme for multiple range-extended target tracking, which results in the development of an optimized LMIPDA algorithm.
(3) We analyze the tracking performance of the proposed algorithm in a sonar underwater scenario.

Our work is organized as follows. In Section 2, a system model for multi-target tracking in noisy jamming environment is discussed. In Section 3, we propose an advanced Doppler measurements association method and extend to the LMIPDA-Doppler measurements association scheme for multi-target tracking in the linear suboptimal framework. In Section 4, the tracking performance of the DDA method is analyzed. The simulation results demonstrating the proposed schemes are presented in Section 5, and conclusions are drawn in Section 6.

## 2. Multiple Target Models

### 2.1. Multiple Target Measurements

The trace of the *k*-th target can be expressed as

(1) $$x_{p+1}^{k}=F_{p}x_{p}^{k}+v_{p}^{k}$$

where $x_{p}^{k}$ is the *k*-th target kinematic state at time p, $F_{p}^{}$ denotes the transition matrix of target state and $v_{p}^{k}$ describes the vector of additive white Gaussian noise (AWGN) with zero mean and covariance $Q_{p}^{k}$. In the Cartesian coordinate system [1], the target kinematic state can be denoted by a vector of six components including position and velocity for each axis $x_{p}^{k}={\left[\begin{array}{cccccc} x_{p}^{} & {\dot{x}}_{p}^{} & y_{p}^{} & {\dot{y}}_{p}^{} & z_{p}^{} & {\dot{z}}_{p}^{} \end{array}\right]}^{\prime }$. During the *p*-th scan, a set of $m_{p}$ sonar observations $Z_{p}=\left\{z_{p,1},z_{p,2},\ldots ,z_{p,m_{p}}\right\}$ are chosen from the system detections. Each observation’s $z_{p,i}^{},i=1,2,\ldots ,m_{p}$ is a vector of four observed values which comprise both range and velocity components from the *i*-th target $z_{p,i}^{}=\left\{y_{p,i}^{c},y_{p,i}^{d}\right\}$. The accumulated measurement sequence up to the *p*-th scan can be denoted as

(2) $$Z_{}^{p}=\left\{Z_{1},Z_{2},\ldots ,Z_{p}\right\}=\left\{Y_{c}^{p},Y_{d}^{p}\right\}.$$

The target position observation has a linear correlation with the target kinematic state. The *k*-th target position observation during the *p*-th scan is denoted as:

(3) $$y_{p}^{c}=H_{p}^{c}x_{p}^{k}+\omega_{p}^{k}.$$

In Equation (3), $H_{p}^{c}=\left[\begin{array}{cccccc} 1 & 0 & 0 & 0 & 0 & 0\\ 0 & 0 & 1 & 0 & 0 & 0\\ 0 & 0 & 0 & 0 & 1 & 0 \end{array}\right]$ denotes the system transition matrix. $\omega_{p}^{k}$ describes the vector of AWGN with zero mean and covariance $R_{p}^{c,k}$. The target velocity measurement from the *k*-th target is denoted as

(4) $$y_{p}^{d}=h\left(x_{p}^{k}\right)+n_{p}^{k}.$$

The measurement error $n_{p}^{k}$ denotes AWGN with zero mean and covariance $R_{p}^{d,k}$. According to the Reference [34], the term $h\left(x_{p}^{k}\right)$ in Equation (4) can be expressed by $h\left(x_{p}^{k}\right)=\frac{\left(x_{p}^{}-x_{p}^{s}\right)\left({\dot{x}}_{p}^{}-{\dot{x}}_{p}^{s}\right)+\left(y_{p}^{}-y_{p}^{s}\right)\left({\dot{y}}_{p}^{}-{\dot{y}}_{p}^{s}\right)+\left(z_{p}^{}-z_{p}^{s}\right)\left({\dot{z}}_{p}^{}-{\dot{z}}_{p}^{s}\right)}{\sqrt{{\left(x_{p}^{}-x_{p}^{s}\right)}^{2}+{\left(y_{p}^{}-y_{p}^{s}\right)}^{2}+{\left(z_{p}^{}-z_{p}^{s}\right)}^{2}}}$, here $x_{p}^{s}={\left[x_{p}^{s},{\dot{x}}_{p}^{s},y_{p}^{s},{\dot{y}}_{p}^{s},z_{p}^{s},{\dot{z}}_{p}^{s}\right]}^{\prime }$ describes the given state vector including sonar position and sonar velocity during the *p*-th scan. We assume that the system process noise $v_{j}^{k}$ and measurement noises $w_{k}^{k}$ and $n_{i}^{k}$ are independent of each other for all j,p,i and k. From position and Doppler measurement models Equations (3) and (4), the conditional probability density functions (PDFs) can be written as

(5) $$\begin{array}{l} p\left(y_{p}^{c}|x_{p}^{k}\right)∼N\left(y_{p}^{c};H_{p}^{c}x_{p}^{k},R_{p}^{c,k}\right)\\ p\left(y_{p}^{d}|x_{p}^{k}\right)∼N\left(y_{p}^{d};h\left(x_{p}^{k}\right),R_{p}^{d,k}\right)\\ p\left(y_{p}^{c},y_{p}^{d}|x_{p}^{k}\right)=p\left(y_{p}^{c}|x_{p}^{k}\right)p\left(y_{p}^{d}|x_{p}^{k}\right)\\ ∼N\left(\left[\begin{array}{c} y_{p}^{c}\\ y_{p}^{d} \end{array}\right];\left[\begin{array}{c} H_{p}^{c}x_{p}^{k}\\ h\left(x_{p}^{k}\right) \end{array}\right],\left[\begin{array}{cc} R_{p}^{c,k} & 0\\ 0 & R_{p}^{d,k} \end{array}\right]\right) \end{array}.$$

### 2.2. Clutter Measurements

During the *p*-th scan, the number of clutter measurements can be considered as an inhomogeneous Poisson distribution. Each clutter component is related to a range measurement and a velocity measurement pair. It is assumed that the target range and velocity measurements of each clutter measurement are independent of each other. The density of each clutter component can be denoted as a product of the PDF of spatial clutter $\rho_{p,i}^{c}$ and the PDF of clutter Doppler $\rho_{p,i}^{d}$ [34]

(6) $$\rho_{p,i}≜\rho \left(z_{p,i}\right)=\rho \left(y_{p,i}^{c}\right)\rho \left(y_{p,i}^{d}\right).$$

It is worth noting that we consider $\rho \left(y_{p,i}^{c}\right)$ and $\rho \left(y_{p,i}^{d}\right)$ as known a priori in this paper.

## 3. Doppler Measurement Association

Based on the LM procedure [31], we develop the DDA method in a joint model for LM tracking for IPDA. To solve the LM tracking problem, we need to estimate the joint posterior density of individual target state $x_{p}^{k}$ conditioned on measurement sequences up to the *p*-th scan $Z_{}^{p}$ as follows

(7) $$p\left(x_{p}^{k},\chi_{p}^{k}|Z_{}^{p}\right),k=1,\ldots ,K.$$

In Equation (7), $\chi_{p}^{k}$ denotes the existence of the *k*-th target at the *p*-th scan. K describes the total number of tracks. We can express the joint posterior density of the track state as follows:

(8) $$p\left(x_{p}^{},\chi_{p}^{}|Z_{}^{l}\right)=p\left(\chi_{p}^{}|Z_{}^{p}\right)p\left(x_{p}^{},|\chi_{p}^{},Z_{}^{l}\right).$$

When l=p, the above formula can be considered as an estimation model. And l=p−1, the above formula is a prediction model. Based on the Bayesian theory, $p\left(x_{p}^{},\chi_{p}^{}|Z_{}^{l}\right)$ can be calculated recursively. At time p, given prior density $P\left(\chi_{p-1}^{}|Z_{}^{p-1}\right)$ and prior density of the target state $p\left(x_{p-1}^{},|\chi_{p-1}^{},Z_{}^{p-1}\right)$, the iterative procedure can be expressed by the following stages.

(1) Calculate the predicted prior density $P\left(\chi_{p}^{}|Z_{}^{p-1}\right)$ and the predicted prior density of the target state $p\left(x_{p}^{}|\chi_{p}^{},Z_{}^{p-1}\right)$.
(2) Measurement selection $Z_{p}=\left\{z_{p,1},z_{p,2},\ldots ,z_{p,m_{p}}\right\}$ via gating for the underlying track.
(3) Calculate the predicted density of the *i*-th target state measurement $p\left(x_{p,i}^{},|\chi_{p}^{},Z_{}^{p-1}\right)$.
(4) Update the posterior density $P\left(\chi_{p}^{}|Z_{}^{p}\right)$ and the posterior density $p\left(x_{p}^{},|\chi_{p}^{},Z_{}^{p}\right)$.

**Figure 1:**
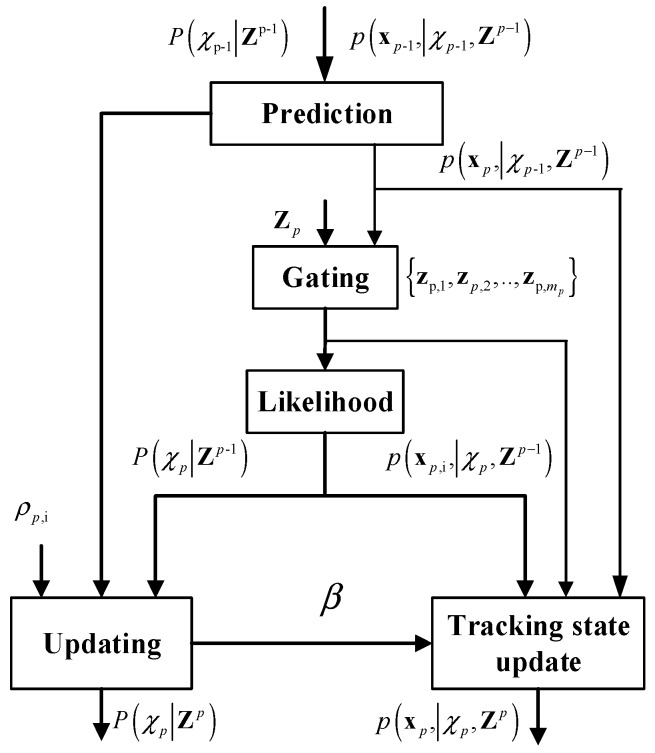
An illustrative diagram for an iteration process of synthetic tracking.

An illustrative diagram for an iteration process of the above four stages is presented in Figure 1.

Step 1: Prediction

Based on the Gaussian hypothesis, every target measurement can be considered as a single Gaussian density function, which is expressed as:

(9) $$p\left(x_{p}|\xi_{p}^{}\left(c\right),\chi_{p}^{},Z_{}^{p-1}\right)∼N\left(x_{p};{\hat{x}}_{p|p-1}\left(\xi_{p}^{}\left(c\right)\right),P_{p|p-1}\left(\xi_{p}^{}\left(c\right)\right)\right)$$

where $\xi_{p}^{}\left(c\right)$ denotes event that the *c*-th out of $C_{p}$ track components is true at the *k*-th sonar scan. ${\hat{x}}_{p|p-1}\left(\xi_{p}^{}\left(c\right)\right)$ and $P_{p|p-1}\left(\xi_{p}^{}\left(c\right)\right)$ describes the mean and covariance of predictive prior density of the target track state, respectively.

Step 2: Measurement Selection

The $Z_{p}=\left\{z_{p,1},z_{p,2},\ldots ,z_{p,m_{p}}\right\}$ measurement selection procedure is implemented at a component level. During the *p*-th scan, the *c*-th of $C_{p}$ components chooses its measurements using a range measurement confirmation gate, which is concentrated at the expected range measurement ${\hat{y}}_{p}^{c}\left(\xi_{p}^{}\left(c\right)\right)$ as follows

(10) $${\left(y_{p,i}^{c}-{\hat{y}}_{p}^{c}\left(\xi_{p}^{}\left(c\right)\right)\right)}^{\prime }{\left[S_{p}^{c}\left(\xi_{p}^{}\left(c\right)\right)\right]}^{-1}\left(y_{p,i}^{c}-{\hat{y}}_{p}^{c}\left(\xi_{p}^{}\left(c\right)\right)\right)\leq \gamma ,$$

where γ denotes a fixed threshold, $y_{p,i}^{c},i=1,\ldots ,m_{p}$ describes the *i*-th confirmed measurement and $S_{p}^{c}{\left(\xi_{p}^{}\left(c\right)\right)}^{2}$ describes the innovation covariance of the *c*-th of $C_{p}$ components. In the gating process, only the range measurements can be utilized to produce a tracking gate for choosing a set of confirmed observations.

Step 3: Predictive Measurement PDF

$p\left(x_{p}^{},|\chi_{p}^{},Z_{}^{p-1}\right)$ is assumed to be a sum of $C_{p}$ mutually exclusive components $\sum_{c=1}^{C_{p}}p\left(\xi_{p}^{}\left(c\right)|\chi_{p}^{},Z_{}^{p-1}\right)p\left(x_{p}^{}|\xi_{p}^{}\left(c\right),\chi_{p}^{},Z_{}^{p-1}\right)$. Consequently, the predicted measurement PDF $\Lambda_{p,i}$ under the assumption of each measurement’s $z_{p,i}^{}$ can be expressed as

(11) $$\begin{array}{ll} \Lambda_{p,i} & ≜p\left(x_{p,i}^{},|\chi_{p}^{},Z_{}^{p-1}\right)\\ & =\sum_{c=1}^{C_{p}}p\left(\xi_{p}^{}\left(c\right)|\chi_{p}^{},Z_{}^{p-1}\right)p\left(y_{p,i}^{c},y_{p,i}^{d}|\xi_{p}^{}\left(c\right),\chi_{p}^{},Z_{}^{p-1}\right) \end{array}.$$

In Equation (11), the term $p\left(y_{p,i}^{c},y_{p,i}^{d}|\xi_{p}^{}\left(c\right),\chi_{p}^{},Z_{}^{p-1}\right)\approx p\left(y_{p,i}^{c}|\xi_{p}^{}\left(c\right),\chi_{p}^{},Z_{}^{p-1}\right)p\left(y_{p,i}^{d}|\xi_{p}^{}\left(c\right),\chi_{p}^{},Z_{}^{p-1}\right)$, which is the measurement likelihood function conditioned on the event $\xi_{p}^{}\left(c\right)$. The joint likelihood can be expressed as a product of target range likelihood and target velocity likelihood.

Step 4: The Probability of Target Existence Update

From the Reference [30], the data association factor can be written as:

(12) $$\delta_{p}=P_{d}P_{g}\left(1-\sum_{i=1}^{m_{p}}\frac{\Lambda_{p,i}}{\rho_{p,i}}\right)$$

where $P_{d}$ and $P_{g}$ denote the probability of detection and the probability of the tracking gate, respectively. $\rho_{p,i}$ denotes the clutter density of a measurement in Equation (6). Then, we can write the probability of target existence as follows:

(13) $$P\left\{\chi_{p}|Z^{p}\right\}=\frac{\left(1-\delta_{p}\right)P\left\{\chi_{p}|Z^{p-1}\right\}}{1-\delta_{p}P\left\{\chi_{p}|Z^{p-1}\right\}}$$

and the data association probabilities can be expressed as:

(14) $$\beta_{p,i}≜\frac{1}{1-\delta_{p}}\{\begin{array}{cc} 1-P_{d}P_{g}, & i=0\\ P_{d}P_{g}\frac{\Lambda_{p,i}}{\rho_{p,i}}, & i>0 \end{array}.$$

It needs to be emphasized that the contribution of target Doppler information is embodied in the factor of data association Equation (12), the probability of target existence Equation (13), and the probabilities of data association Equation (14).

Step 5: Tracking Update

From the Reference [30], the prior density of the target state is updated as follows:

(15) $$\begin{array}{l} p\left(x_{p}^{},|\chi_{p}^{},Z_{}^{p}\right)\\ =\sum_{i=0}^{m_{k}}\sum_{c=1}^{C_{p}}P\left\{\xi_{p}\left(c\right)|\chi_{p}^{},Z_{}^{p-1}\right\}\beta_{p,i}\frac{\Lambda_{p,i}\left(\xi_{p}\left(c\right)\right)}{\Lambda_{p,i}}p\left(x_{p}^{},|\xi_{p}\left(c\right),\chi_{p}^{},z_{p,i},Z_{}^{p}\right)\\ =\sum_{c=1}^{C_{p+1}}p\left(\xi_{p+1}\left(c\right)|\chi_{p}^{},Z_{}^{p}\right)p\left(x_{p}^{},|\xi_{p+1}\left(c\right),\chi_{p}^{},Z_{}^{p}\right) \end{array}.$$

The term $p\left(x_{p}^{},|\xi_{p}\left(c\right),\chi_{p}^{},z_{p,i},Z_{}^{p}\right)$ in (15) is the conditional posterior density, which can be denoted as

(16) $$p\left(x_{p}^{},|\xi_{p}\left(c\right),\chi_{p}^{},z_{p,i},Z_{}^{p}\right)=\frac{p\left(y_{p,i}^{c}|x_{p}\right)p\left(y_{p,i}^{d}|x_{p}\right)p\left(x_{p}|\xi_{p}\left(c\right),\chi_{p}^{},Z_{}^{p-1}\right)}{p\left(y_{p,i}^{c}|\xi_{p}\left(c\right),\chi_{p}^{},Z_{}^{p-1}\right)p\left(y_{p,i}^{d}|\xi_{p}\left(c\right),\chi_{p}^{},Z_{}^{p-1}\right)}.$$

Because of the nonlinear relationship between target state and target Doppler $h\left(x_{p}^{k}\right)$, we have to employ a nonlinear filter for solving Equation (16). In our work, in order to simplify the discussion, we choose not to use the Doppler measurements track state updates. The term $p\left(y_{p,i}^{d}|x_{p}\right)$ can be approximated as $p\left(y_{p,i}^{d}|\xi_{p}\left(c\right),\chi_{p}^{},Z_{}^{p-1}\right)$. Therefore, the conditional posterior density can be rewritten as

(17) $$p\left(x_{p}^{},|\xi_{p}\left(c\right),\chi_{p}^{},z_{p,i},Z_{}^{p}\right)=N\left(x_{p}^{}\left(\xi_{p}\left(c\right)\right);x_{p|p}^{c,j},P_{p|p}^{c,j}\right).$$

It is worth noting that the conditional posterior density becomes the standard Kalman filter form. That is to say, $p\left(x_{p}^{},|\xi_{p}\left(c\right),\chi_{p}^{},z_{p,i},Z_{}^{p}\right)$ represents the output of a standard Kalman filter with predicted target state ${\hat{x}}_{p|p-1}\left(\xi_{p}^{}\left(c\right)\right)$ and covariance $P_{p|p-1}\left(\xi_{p}^{}\left(c\right)\right)$ as stated in step 1.

Considering the problem of multi-target tracking, the LM scheme views all observations from other targets as clutter. Therefore, the density of clutter can be modulated by contributions from other targets. During the *p*-th scan, the LM scheme estimates a revised density of clutter for all tracking gates, which is employed to compute the data association factor, the target existence probability and measurement association probabilities for all K targets. As a result, the probability $P_{i}^{k}$ that the *i*-th measurement related to the *k*-th target can be expressed as

(18) $$\begin{array}{l} P_{i}^{k}=P\left(\theta_{p,i}^{k},\chi_{p}^{k}|Z_{}^{p-1}\right)\\ =P_{d}^{k}P_{g}^{k}P\left(\chi_{p}^{k}|Z_{}^{p-1}\right)\frac{\Lambda_{p,i}^{k}}{\sum_{i=1}^{m_{p}}\Lambda_{p,i}^{k}} \end{array}.$$

In Equation (18), $\theta_{p,i}^{k}$ denotes the event that the *i*-th measurement is resulted from the *k*-th track at time p. $\Lambda_{p,i}^{k}$ is presented in Equation (11). $P\left(\chi_{p}^{k}|Z_{}^{p-1}\right)$ describes the predicted prior probability of the *k*-th target. Owing to the presence of multi-target, the modified clutter density $\Omega_{p,i}^{k}$ in the gate of the *k*-th track can be expressed as:

(19) $$\Omega_{p,i}^{k}=\rho_{p,i}^{}+\sum_{\delta =1,\delta \neq k}^{K}\Lambda_{p,i}^{\delta }\frac{P_{i}^{\delta }}{1-P_{i}^{\delta }}.$$

Hence, relying on a linear multi-target scheme, we can obtain the data association factor of the *k*-th target at time p as follows:

(20) $$\delta_{p}^{k}=P_{d}^{k}P_{g}^{k}\left(1-\sum_{i=1}^{m_{p}^{k}}\frac{\Lambda_{p,i}^{k}}{\Omega_{p,i}^{k}}\right).$$

The measurement association probability of the *k*-th target at time p can be expressed as

(21) $$\beta_{p,i}^{k}=\frac{1}{1-\delta_{p}^{k}}\{\begin{array}{cc} 1-P_{d}^{k}P_{g}^{k}, & i=0\\ P_{d}^{k}P_{g}^{k}\frac{\Lambda_{p,i}^{k}}{\Omega_{p,i}^{k}}, & i>0 \end{array}.$$

Therefore, LM-IPDA scheme with DDA can be acquired. The DDA method can be used in the joint IPDA algorithm for target tracking in clutter. The joint IPDA algorithm recursively updates both the probability of target existence and target state estimate. The probability of target existence is used as a track quality measure for false track discrimination. It is worth noting that the contribution of multi-target Doppler information is also embodied in the factor of data association Equation (20), the probability of target existence Equation (18), and the probabilities of data association Equation (21). 

## 4. Performance Evaluation

During the *p*-th scan, the current tracks, which can be described by mean ${\hat{x}}_{p|p}^{}$ and covariance $p_{p|p}^{}$, are updated by using the current observations. All observations, which lie outside the validation gates ${\left(y_{p,i}^{c}-{\hat{y}}_{p}^{c}\right)}^{\prime }{\left[S_{p}^{c}\right]}^{-1}\left(y_{p,i}^{c}-{\hat{y}}_{p}^{c}\right)>\gamma$, are considered as irrelevant observations. Any irrelevant observation is processed by the track initiation module, where tentative tracks are formed based on the observations from two successive scans, that is, a window spanned by the maximum expected target velocity over a scan period selects all possible pairs of observations from two successive scans. Consequently, the observation pairs form tentative tracks by using the two-point initiation technique [35].

An initial target existence probability $P\left\{\chi_{p}|Z^{p}\right\}$ will be assigned to all new tracks. All tracks can be updated recursively by using a new measurement set, the corresponding probabilities of target existence are updated too. During the tracking procedure, the probabilities of target existence are evaluated against specific thresholds for track confirmation and termination. A track exists if its probability of target existence is greater than the predefined threshold of track confirmation. A track disappears if its probability of target existence is below the predefined threshold of track termination. Multiple tracks, which are close to each other, would be combined into a single track. Only the confirmed tracks are displayed to the observer. We evaluate multi-target tracking performance based on the three criteria as follows:

A. Number of confirmed true tracks (NCTT) and Number of confirmed false tracks (NCFT)

A track in correspondence of the true state of a target can be considered as a true track. The *j*-th track denoted by mean ${\hat{x}}_{p|p}^{j}$ and covariance $p_{p|p}^{j}$ has relation with the *j*-th target state $x_{p}^{j}$, a constant threshold ψ is employed to satisfy ${\left({\hat{x}}_{p|p}^{j}-x_{p}^{i}\right)}^{\prime }{\left(p_{p|p}^{j}\right)}^{-1}\left({\hat{x}}_{p|p}^{j}-x_{p}^{i}\right)\leq \psi$. On the contrary, a track that does not associate with any true target state can be viewed as a false track. NCTT and NCFT are important measures for the multi-target tracking performance.

B. Target resolution

A possible issue with target state measure is that a track related to closely spaced targets would be viewed as one true track. Assuming the sampling frequency $f_{s}$, the carrier frequency $f_{c}$ and the speed of electromagnetic wave c, the range resolution is $\Delta R≜\frac{c}{2f_{s}}$ and the velocity resolution is $\Delta v≜\frac{c\Delta f_{d}}{2f_{c}}$, where $\Delta f_{d}$ denotes the Doppler shift resolution. We use mean square error (MSE) performance to define target resolution.

C. The ability to capture the target

The measure calculates the frequency that a confirmed track associates with an observable true target state. We use MSE performance to define the ability of capturing the target. 

## 5. Simulation

The performance enhancement of multi-target tracking provided by the proposed DDA scheme with respect to different criteria is demonstrated in this section. We compare the tracking performance of the LMIPDA algorithm using the DDA scheme with nonlinear filtering method mentioned in [12] in a sonar underwater target tracking scenario.

### 5.1. Sonar Underwater Tracking

We propose the underwater scenario as follows: A warm water surrounding with depth (120 m) and acoustic velocity (1460 m/s) was employed for the simulated experiment. Sound transmission was represented by using multipath expansions with propagation properties for instance water refraction, attenuation and spreading as presented in [36]. The proposed system was denoted by an active sonar array, which was appropriate for transmitting and receiving short wave sound signals. The signal transferred 1/3 second pulses, each 5 s over an 8 min period. Transmit signals were composed of either continuous wave (CW) or phase modulated pulse waveforms that signified two diverse location and velocity resolutions. Generally, the continuous waveform had improved velocity resolution and worse location resolution than the phase modulated pulse waveforms. In theory, the CW waveforms have a location resolution (220 m) and velocity resolution (0.1 m/s) while the phase modulated pulses have location resolution (0.6 m) and velocity resolution (10 m/s). We considered that the target Doppler was limited to the interval [−20, +20] m/s to make allowances for the target speeds up to 30 knots. The proposed sonar systems were able to detect multiple targets and ranges in the interval [220, 4200] m.

There are ten targets in following scenario, including four range-spread targets and six point targets. The range-spread target is considered as a linear time-invariant filter with random impulse response, where the amplitude of each range cell is a zero-mean Gaussian variable. Target kinematic state and target position state are modeled by Equations (1) and (3), respectively. The transition matrix of target kinematic state $F_{p}$ and the covariance of the noise $Q_{p}$ can be described as follows

(22) $$F_{p}=diag\left(F_{1},F_{1},F_{1}\right),F_{1}=\left[\begin{array}{cc} 1 & T\\ 0 & 1 \end{array}\right]$$

and

(23) $$Q_{P}=diag\left(Q_{1},Q_{1},Q_{1}\right),Q_{1}=\left[\begin{array}{cc} T^{3}/3 & T^{2}/2\\ T^{2}/2 & T \end{array}\right]$$

where T denotes data sampling interval for tracking. The sampling interval of transmit impulse was 5 s. The parameter T varied owing to a limited acoustic transmission period and time-variant ranges of multiple targets. We considered the noise parameter of the proposed system process was q=0.1. The initial parameters of all targets are presented in Table 1.

**Figure 2:**
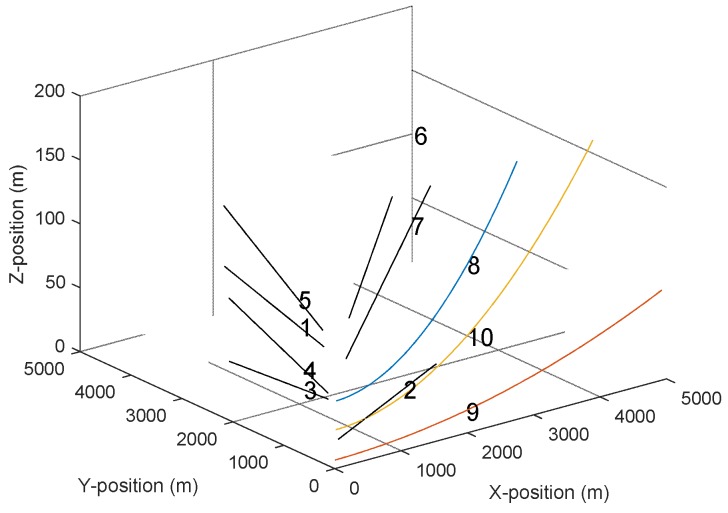
The 3D trajectory of ten targets diagram.

We considered the targets 5–10 had constant velocity and the targets 1–4 were in accelerated motion. The range-spread submarine was characterized by a cluster of ten scattering points organized in a given form of length 60 m that was corresponding to the heading of the object. Similarly, the range-spread larger ship was characterized by a cluster of thirteen scattering points organized in a given form of length 100 m. The three-dimensional (3D) track trajectory of all targets is shown in Figure 2.

The transmit waveform illuminated sonar targets, which was reflected by certain targets. The backscattering signals were received by the proposed system. A cluster of transmission pathways comprising three bounces, which were calculated in every direction gave rise to 185 paths for every scattering point. We modeled all objects as a point scatter or a series of point-like targets.

A sonar signal processing method, which changes with waveform mode, can be utilized to calculate the response of the proposed transceiver to the whole set of sonar echoes. With the approach, a clustering procedure integrates all sonar echoes that fall within the similar sonar resolution cell into a particular cluster of sonar echoes. The approximation errors for the clustered sonar echoes can be calculated from the target scattering signal power, the interference noise characteristics and the measurement resolution, and are much smaller than the related measurement resolution. In our work, the approximation errors of the range and Doppler for both CW and phase modulated pulse mode are presented in Table 2.

The measurement errors of the bearing and elevation were about 2 deg for the two transmit types. It is worth noting that the clustering procedure increased the measuring errors on the basis of the number of the clustered sonar echoes and spread in measurement space. It influenced the range measurements the most because of signal echoes received at the sonar system along a multipath with a similar the frequency domain but a relative spread in the time domain.

The measured values of range, bearing and elevation can be mapped into Cartesian location values [37]. The clustering procedure integrated sets of multiple target detections at a distance of 12 m from each other into a clustered detection. The multipath from a range-spread target to the proposed system gave rise to false images looking like a cluster of reproductions below the water bottom or above the water surface. For the aims of multi-target tracking research, every clustered observation which size is larger than 15 m below water bottom can be excluded to eliminate the numerous images of the sonar target. We used the additional target-clustering procedures in combination with the filtering of detections to solve the problem of multiple target detections. However, multiple target images were still produced for a point target or the spatially spread scattering points corresponding to a range-spread target because of the multipath from the sonar system to the target. 

The detection threshold can be deduced from the corresponding false alarm probability, which relates to the clutter quantitative value. For a given false alarm probability $p_{fa}=0.0001$, the expected value of clutter measurements varied with the number of measuring resolution cells. There were 120 clutter detections per scan.

**Figure 3:**
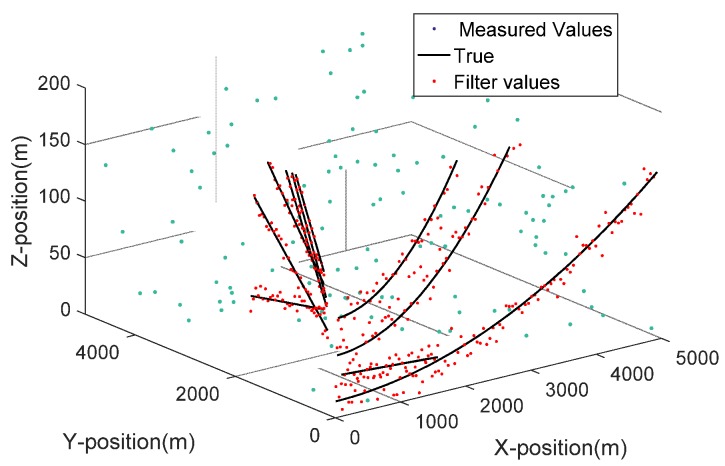
The target position measurements.

**Figure 4:**
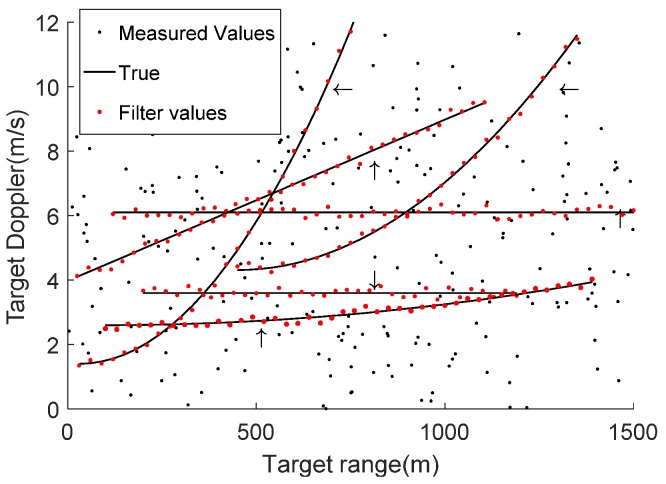
Doppler measurements versus range for continuous wave (CW) mode.

The target position measurements are shown in Figure 3. Figure 4 presents Doppler measurements versus range for CW mode. The multiple target detections in a sea clutter environment were observed by the sonar system over 100 scans for a single simulated experiment. There were two possibilities. The first was that the target lay outside the proposed system’s field of view. The second was that the received signal strength was lesser than the given detection threshold. Not all targets were clearly observed during each sonar scan. Multiple target detections were not perceived for targets 1 and 2 during certain parts of the simulated experiment. The range-spread larger target 3 approached the dim point target 4 during the 38th–44th sonar scan and approached two stationary point targets 5 and 6 to offer a sonar sea multi-target tracking environment.

### 5.2. Performance Evaluation

The simulation parameters associated with the proposed schemes, comprising the initial target existence probability, track confirmation threshold and track termination threshold were assumed to obtain the greatest capability. These experiment parameters are shown in Table 3.

The results were obtained from 600 Monte Carlo simulations for both transmit modes. During each simulation run, three trackers (the extended Kalman filtering technique, LMIPDA, LMIPDA-DDA) were applied to the same measurement data employing three schemes: (1) Updating tracks via target range measurements based on extended Kalman filtering as presented in [12], (2) updating tracks via target range measurements based on the regular trackers LMIPDA, and (3) updating tracks via both target range measurements and the supplementary target velocity measurements based on the advanced trackers LMIPDA-DDA. 

**Figure 5:**
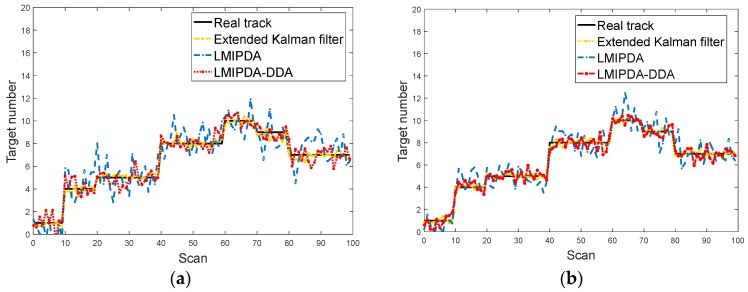
The performance of the number of confirmed true tracks (NCTT): (**a**) CW mode; (**b**) pulse mode.

The comparisons of NCTT across three methods are illustrated in Figure 5a,b. The average computation time is presented in Table 4.

As shown in Figure 5 and Table 4, the LMIPDA-DDA algorithm had an enhanced confirmation response to multi-target observations. Meanwhile, the accuracy of NCTT provided by the LMIPDA-DDA algorithm was obviously superior to the LMIPDA algorithm. The LMIPDA method played an important part on single scan tracking but was not robust enough for abrupt changes in target tracking, while the LMIPDA-DDA method played an important part on multiple scan tracking. The extended Kalman filtering method as stated in [12] was slightly better than the proposed DDA scheme. The nonlinear filtering technique was optimal in this case.

As shown in Table 4, a faster calculating speed can be obtained. With Doppler data association, the average operation times of the LMIPDA-DDA algorithm were lower. Furthermore, the top operation times of the LMIPDA-DDA algorithm were also lower than other methods due to data association. Faster termination of false tracks can be observed. As can be expected, the nonlinear filtering technique spent the most time to implement iterated operation. However, if the distribution of target velocity component is hard to distinguish from the distribution of false target velocity component, the tracking capability of data association method would reduce to that of the traditional method. It is worth noting that if the proposed system switched between CW and phase modulated pulse transmission modes, the measurement errors of target range and Doppler would vary as presented in Table 2. The errors would undoubtedly impact the multi-target tracking performance under consideration. To simplify the discussion, the problem has been ignored in this paper.

**Figure 6:**
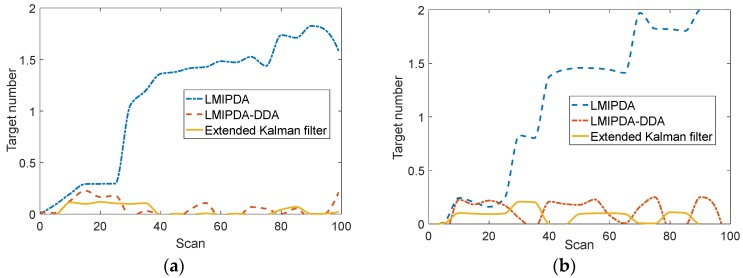
The performance of the number of confirmed false tracks (NCFT): (**a**) CW mode; (**b**) pulse mode.

The comparison of NCFT across methods is shown in Figure 6. As can be seen from Figure 6, a remarkable reduction in NCFT was seen for both CW and pulse modes. The presence of confirmed false tracks can severely limit confidence level and usage of trackers; better discrimination of false tracks allows for reduction of track confirmation threshold, which leads to better response to target measurements. When the transmitter was switched to pulse mode, target Doppler measurements got larger errors as present in Table 2. As can be expected, a remarkable reduction in NCFT was still obtained from Figure 6b. That is because the distribution of target Doppler measurements could be discriminated from that of false Doppler measurements. 

**Figure 7:**
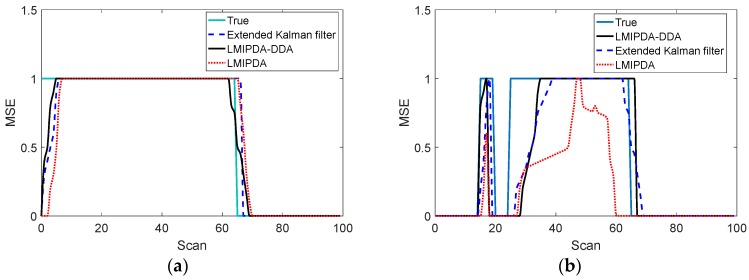

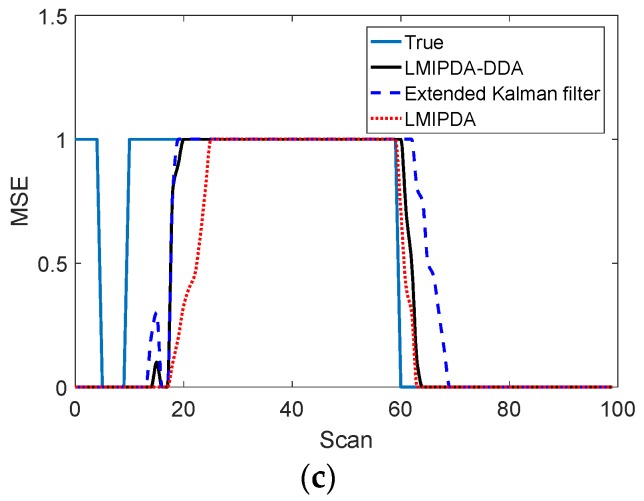
The capabilities of capturing targets 1–3 in CW mode; (**a**) target 1; (**b**) target 2; (**c**) target 3.

**Figure 8:**
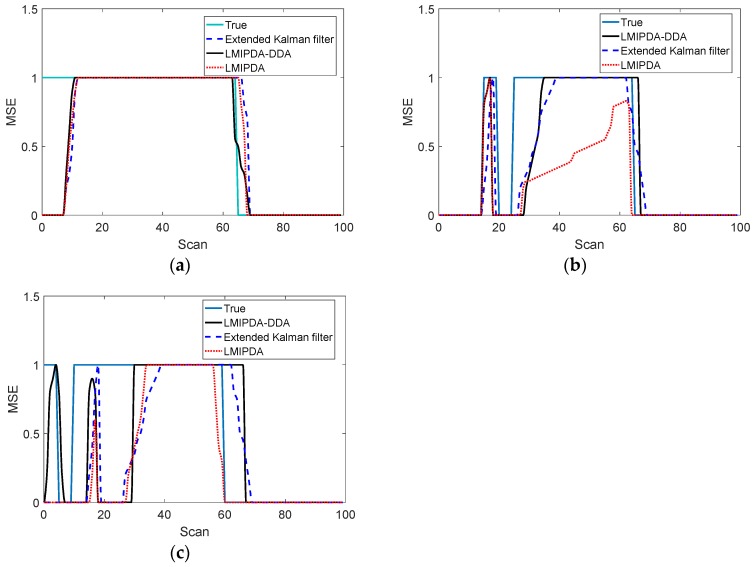
The capabilities of capturing targets 1–3 in pulse mode; (**a**) target 1; (**b**) target 2; (**c**) target 3.

The comparison of the ability of capturing the target across methods is presented in Figure 7 and Figure 8. Performance difference for targets 1–3 is given in this section. The rest of targets show a similar trend. The proposed DDA method had a better confirmation response to target measurements in most cases.

As can be seen from Figure 7c, during the first 10 scans, only the proposed DDA method had captured the underlying target 3. We can explain the phenomenon by understanding that the LMIPDA-DDA algorithm works on single scan measurements, while the extended Kalman filtering technique stated in [12] works on multiple scan measurements. Hence, the former will be more adaptable to abrupt changes in target measurements.

Furthermore, as shown in Figure 8, the proposed DDA method and the extended Kalman filtering technique suffered from a longer true track confirmation delay compared with the CW mode case, as presented in Figure 7, because target information flow rate from the target measurements was lower. It is easy to see that, if the distribution of target Doppler measurements is indistinguishable from the distribution of false Doppler measurements, the performance of the trackers with DDA method would reduce to that of the trackers without DDA method.

**Figure 9:**
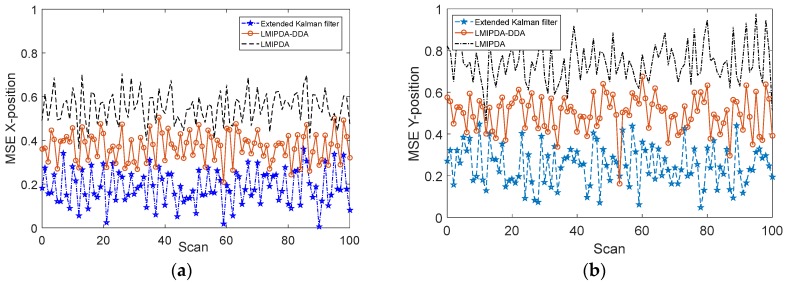
The comparisons of mean square error (MSE) performance across methods: (**a**) MSE in X-axial; (**b**) MSE in Y-axial.

The comparisons of target resolution capability across methods are given in Figure 9a,b.

As can be seen from Figure 9a,b, the target resolution capability offered by the LMIPDA-DDA algorithm was better than the LMIPDA algorithm at each scan. However, as can be expected, the performance provided by the nonlinear filtering technique was slightly better than the proposed DDA scheme. The simulation result demonstrated that, compared with the traditional tracking algorithm without DDA, the resolution capability of the sensor system provided by the LMIPDA-DDA algorithm was obviously improved. The key difference between the Doppler measurement association scheme and traditional methods without DDA was that in the Doppler measurement association scheme, the target velocity observed values were used for measurement association. The sonar sensor system improved by Doppler data association scheme had little influence on its operations in terms of system robustness and filter complexity but offered a significant decrease in the amount of false observations. The computational complexity was also greatly reduced. Therefore, if we make a trade-off between complication and performance gain, the proposed DDA scheme was superior to nonlinear filtering technique in this case.

## 6. Conclusions

In this paper, a sonar sensor system provided by a Doppler measurement association method was proposed for enhancing the performance of multi-target tracking in a noisy jamming environment. In the Doppler measurement association scheme, the target velocity measurements are utilized for calculating the observation likelihood, which are an important part for distinguishing true measurements from phony targets or clutter measurements. The sonar system improved by the proposed scheme has a tiny influence on system stability but offers a significant decrease in the amount of confirmed false targets. Meanwhile, the target resolution capability of the system provided by the LMIPDA-DDA algorithm is obviously improved, which can be realized without equipping a nonlinear filter bank. In the clutter environment, the traditional method that uses location-only component obtain a substantial NCTT, while the proposed method that combine target velocity components do not. The usefulness of the enhanced DDA scheme has been verified in a sea clutter environment. 

## Figures and Tables

**Table 1:** The initial parameters of all targets.

Target	Category	Location	Velocity	Acceleration	Azimuth
1	Submarine	[−400, 200, −30]	8.6 m/s	4.5 m/s^2^	51.3 deg
2	Submarine	[−600, 300, −50]	11.6 m/s	5.3 m/s^2^	200.3 deg
3	Surface ship	[−1200, 1300, 0]	7.6 m/s	3.2 m/s^2^	150.2 deg
4	Small boat	[−650, 450, 0]	9.6 m/s	3.4 m/s^2^	
5	Point buoy	[−150, 800, −20]	3.6 m/s	0	
6	Point buoy	[−890, 1400, −20]	3.6 m/s	0	
7	Point buoy	[−90, 30, −100]	3.6 m/s	0	
8	Point bottom	[−920, 890, −100]	6.1 m/s	0	
9	Point bottom	[−1920, 1890, −100]	6.1 m/s	0	
10	Point bottom	[−80, 90, −100]	6.1 m/s	0	

**Table 2:** Measurement error.

Transmit Types	Range Error	Doppler Error
CW	95.3 m	0.025 m/s
Phase modulated pulse	0.85 m	8.5 m/s

**Table 3:** Tracker parameter settings.

Simulation Parameters	Extended Kalman Filtering	LMIPDA	LMIPDA-DDA
Thresholds for track confirmation	0.95	0.95	0.95
Thresholds for track termination	0.005	0.005	0.005
Merge threshold	4	4	4
Initial probability of target existence	0.08	0.08	0.08
Detection probability	0.98	0.98	0.98
System process noise	0.1	0.1	0.1
Process noise for Doppler state	1 m/s	1 m/s	1 m/s
Target speed	800 m/s	800 m/s	800 m/s

**Table 4:** The average computation times in CW and pulse transmission methods.

Method	NCTT	CPU	Peak CPU
**CW mode**			
LMIPDA	1.245	1.545	5.142
LMIPDA-DDA	0.0041	1.301	3.978
Extended Kalman filtering	4.643	8.183	9.112
**Pulse mode**			
LMIPDA	1.345	1.312	4.265
LMIPDA-DDA	0.001	1.532	3.532
Extended Kalman filtering	3.053	6.423	9.023
